# Use of Nuclear Factor of Activated T Cell-Regulated Gene Expression for Monitoring Immunosuppression with Extended-Release Tacrolimus after Liver Transplantation—A Proof of Concept

**DOI:** 10.3390/pharmaceutics16101317

**Published:** 2024-10-11

**Authors:** Judith Kahn, Eva Maria Matzhold, Peter Schlenke, Peter Schemmer

**Affiliations:** 1 Division of General, Visceral, and Transplant Surgery, Department of Surgery, Medical University of Graz, 8036 Graz, Austria; 2 Department of Blood Group Serology and Transfusion Medicine, Medical University of Graz, 8036 Graz, Austria; eva.matzhold@medunigraz.at (E.M.M.); peter.schlenke@medunigraz.at (P.S.); 3 Bern Visceral Surgery & Pancreas Clinic Switzerland, Hirslanden Hospital Beau-Site, 3013 Bern, Switzerland

**Keywords:** immunomonitoring, nuclear factor of activated T cells (NFAT), residual gene expression (RGE), extended-release Tacrolimus (LCP Tac), liver transplantation

## Abstract

Background: There is a narrow therapeutic window for immunosuppression using calcineurin inhibitors. Drug trough levels do not reflect immunosuppression and should be replaced by pharmacodynamic monitoring. This prospective cohort study was designed to evaluate the effect of an extended-release formulation of tacrolimus (LCP Tac) on the nuclear factor of activated T cell-regulated gene expression (NFAT-RGE). Methods: The expression of interleukin-2, interferon-γ, granulocyte-macrophage colony-stimulating factor, and three reference genes was measured. Samples from 23 patients at defined time points in the first year after liver transplantation were analyzed using a droplet digital polymerase chain reaction. Results: All samples were within the targeted trough levels of LCP Tac, and their LCP Tac peak levels and residual NFAT-RGE showed a strong inverse correlation (r = −0.8). Most importantly, there was an individual immunosuppressive response to the LCP Tac. The mean individual trough effect of LCP Tac on the three target genes when all time points were pooled was 33% (26–56%) in patients without infection and 81% (53–95%) in those with infection (*p* < 0.011). The mean individual peak effect was 48% (44–64%) in patients without infection and 91% (90–94%) in those with infection (*p* < 0.001). Conclusions: Thus, tailored immunosuppression based on residual NFAT-RGE could prevent infections associated with over-immunosuppression early after liver transplantation.

## 1. Introduction

Calcineurin inhibitors (CNIs) are the backbone of immunosuppression (IS) following liver transplantation (LT); however, treatment with CNIs has many undesirable side effects. Although they are dose-critical drugs with a narrow therapeutic range, adequate monitoring is not currently available. As defined serum CNI trough levels have an individual effect on the immune status of each patient, the current standard for their monitoring does not reflect the overall immunological effect of CNIs. Thus, there is an urgent need to directly monitor the effects of CNIs for tailored IS and to reduce the side effects, such as infections, of these drugs. Careful pre- and post-transplant screening, vaccination pre- and post-LT, and post-LT prophylactic antimicrobials may reduce the risk of infection, which is currently the leading cause of death in these patients. Opportunistic pathogens, such as cytomegalovirus (CMV), which are not harmful to immunocompetent individuals, are predominantly responsible for this process. In general, IS increases the risk of infection, with the highest prevalence in over-immunosuppressed patients.

Recent evidence suggests that IS can be measured using surrogate markers, such as the Torqueo Teno virus and polyoma (BK) virus load [1,2]. Additionally, CNIs inhibit the phosphatase activity of calcineurin after the formation of CNI/calmodulin complexes in the cytosol of lymphocytes. The main substrate of calcineurin in T cells is the phosphorylated transcription factor, the nuclear factor of activated T cells (NFAT). The dephosphorylation of NFAT by calcineurin is required for the translocation of this transcription factor into the nucleus, allowing for the subsequent transcription of the four key genes needed for T cell activation: interleukin-2 (IL-2), tumor necrosis factor alpha, granulocyte-monocyte colony-stimulating factor (GM-CSF), and interferon-gamma (IFN-γ) [3]. The expression of the three NFAT-regulated genes, IL-2, IFN-γ, and GM-CSF, can be analyzed to directly measure the actual functional effects of CNIs in peripheral blood [4,5]. Using a real-time polymerase chain reaction (PCR), several studies have semi-quantitatively analyzed NFAT-regulated gene expression (RGE) in peripheral blood and have confirmed this approach to be useful for the individualization of CNI dosing. Nearly all studies have shown an association between the level of NFAT-RGE and clinical aspects, which include rejection episodes and complications such as infection and malignancy.

The present study is the first designed to analyze the individual effects of Envarsus^®^ (LCP Tac), a once-daily extended-release Tac formulation with MeltDose™ drug-delivery technology used to control drug release and enhance the drug’s overall bioavailability, on the expression of NFAT-RGE. The use of LCP Tac in immunosuppressed patients following kidney transplantation (KT) and LT can potentially increase bioavailability, lower blood peak levels (c_max_), lower peak-to-trough fluctuations, allow for the faster achievement of therapeutic drug levels, and lower the total daily dose when compared to Prograf^®^ and Advagraf^®^. Compared to Prograf^®^, an increase in the concentration/dose ratio has been shown to be associated with improvements in renal function in LT patients, with a significant recovery of their estimated glomerular filtration rate 6 months after conversion [6]. When compared to immediate-release Tac, a lower incidence of tremor, insomnia, and alopecia after conversion with LCP Tac in KT patients within the first year post-transplant was also shown [7]. The aim of this trial is to prove the concept that this diagnostic approach can be used to monitor the degree of IS in LT patients receiving LCP Tac.

## 2. Materials and Methods

### 2.1. Patients

This prospective observational cohort study included 23 patients who underwent an LT between February 2019 and June 2020 (phase IV). They were recruited at the Division of General, Visceral, and Transplant Surgery, Department of Surgery, Medical University of Graz, Austria, and were investigated before LT (pre-LT) and at 1 and 2 weeks and 1, 6, and 12 months after LT (post-LT). Measurements were also performed at the time of infection. Infection requiring antimicrobial treatment was defined as exhibiting leukocyte and C-reactive protein values above the normal range, as confirmed by cell culture and viral PCR results, when possible. The diagnosis and treatment of infections were routinely performed. Rejection was primarily diagnosed through clinical assessments and liver function tests, i.e., elevated liver enzymes, after ruling out vascular and biliary complications using imaging techniques. If there was an immediate response to the administration of corticosteroids, a liver biopsy was not performed [8].

Briefly, post-LT patients who provided informed consent and were at an age ≥ 18 and <90 years were included in this study. Patients with a known allergy to the study drug, or its constituents, or to medications with a similar chemical structure, as recently published, were excluded from this study (NCT03315858).

#### Immunosuppressive Treatment

The IS regimen used in this study consisted of LCP Tac, which was introduced directly after LT, with mycophenolate mofetil and corticosteroid tapering for 2 months. Induction therapy with anti-T-lymphocyte globulin (ATG) at 3–5 mg/kg for 4 days was administered to patients <40 years of age with autoimmune hepatitis, with renal insufficiency with a glomerular filtration rate of < 60 mL/min, and those receiving grafts from donors after cardiac death. All patients received the extended-release, once-daily formulation of LCP Tac (Envarsus^®^, Chiesi Farmaceutici S. p. A., 43122 Parma, Italy). The initial oral once-daily dose of LCP Tac was 0.11–0.13 mg/kg, initiated within 24–72 h after surgery. This dose was subsequently adjusted to achieve target trough serum concentrations of 5–10 ng/mL [8]. Their LCP Tac serum levels were also measured 4 h after LCP Tac intake (LCP Tac_peak_). A total of 1 g of mycophenolate mofetil was administered twice daily. Steroid treatment consisting of 500 mg of methylprednisolone was initiated on day 0, with 160 mg administered on day 1, 80 mg on day 2, and 40 mg from day 3 onward. The dose was progressively decreased to allow patients to be weaned off and discontinue steroids after postoperative day (POD) 60.

Patients with CMV-positive donors received CMV prophylaxis, which included valganciclovir during the first 6 months post-LT. Both CMV-positive and -negative recipients received CMV prophylaxis with valganciclovir during the first 3 months post-LT.

LCP Tac levels were determined by liquid chromatography with tandem mass spectrometry (LC-MS/MS) using ethylendiamintetraacetate (EDTA) anticoagulated blood and a validated CE IVD assay (MassTox^®^ Immunosuppressants in whole blood, Chromsystems).

### 2.2. Gene Expression Analysis

Heparinized peripheral blood samples (4 mL) were stimulated with 4 mL of complete RPMI-1640 medium (#R8755) containing 5 µg/mL Ionomycin (#I3909) and 100 ng/mL PMA (P1585) (all from Sigma-Aldrich, Burlington, MA, USA). After incubation at 37 °C for 3 h, lysates of the stimulated cells were prepared using Buffer EL (#79217) and RLT (#79216), and their total RNA was isolated using the RNA Blood Mini Kit (#52304) (all from Qiagen). Complementary DNA (cDNA) was reverse-transcribed using the SuperScript III First-Strand Synthesis System for RT-PCR (#18080051) (Invitrogen, Life Technologies), according to the manufacturer’s protocols. Reactions consisting of 0.15–1.5 ng of cDNA were used in droplet digital PCR (ddPCR) experiments.

The gene expression levels of IL-2, IFN-γ, GM-CSF, and 3 reference genes, ACTB, GAPDH, and TPT1, were quantified using ddPCR (QX200 Droplet Digital PCR System) with the ddPCR Supermix for Probes (#186-3023) (Both from Bio-Rad, Hercules, CA, USA). Human ddPCR Expression Probe Assays—dHsaCPE5192847 (IL-2), dHsaCPE5034618 (IFNG), and dHsaCPE5039652 (CSF2)—were multiplexed with dHsaCPE5190200 (ACTB), dHsaCPE5031067 (TPT1), and dHsaCPE5031597 (GAPDH), respectively. The absolute number of copies (c) of the target gene transcripts were normalized to the geometric mean of the absolute copies of the 3 reference genes.

At 1 and 2 weeks and 1, 6, and 12 months after LT, the residual expression of NFAT-RGE after an LCP Tac intake was calculated. This percentage (RGE%) was calculated as c_peak_/c_0_ × 100, with c_0_ as the number of transcripts at the LCP Tac pre-dose level and c_peak_ as the number of transcripts at the LCP Tac peak level, 4 h after the LCP Tac intake according to whole-blood time–concentration curves observed in stable transplant recipients [9,10].

The peak effect of LCP Tac (Effect LCP Tac_peak_) on gene expression was determined as c_peak_ for the patients’ respective visits, which was adjusted to the pre-LT copy number (c_pre-LT_). The trough effect of LCP Tac (Effect LCP Tac_0_) was determined as c_0_ for their respective visits, adjusted to the c_pre-LT_. [11]. The pooled gene expression was calculated as the average of all copies (IL-2, GM-CSF, and IFN-γ) for each visit, and the mean copies and RGE% were calculated as the mean value over all eligible time points. The median of the pooled values of all patients without infection was compared with the median of the pooled values of all patients with infection.

### 2.3. Statistical Analysis

Patient characteristics and laboratory data are described as medians and interquartile ranges and counts and proportions, respectively. The study was planned to have a significant correlation of +/− 0.65 with a power of 90% and a 2-sided alpha of 0.05. To assess statistical differences between groups, U-tests based on Mann–Whitney and Wilcoxon’s methods were performed. To assess differences between time points, Friedman or Wilcoxon signed-rank tests were used. All reported *p*-values were two-sided, with *p* ≤ 0.05 considered statistically significant. Additionally 25 and 75 Percentiles were calculated (q1, q3). Spearman’s correlation coefficient (ρ) was used to quantify bivariate relationships. A ρ > 0.7 was considered to indicate a strong relationship. The time to infection was described using the Kaplan–Meier method. Statistical analyses were performed using SAS 9.4 software.

## 3. Results

A total of 23 patients (65.2%, n = 15 male, n = 8 females) with a mean age of 59 underwent LT for either alcoholic liver cirrhosis (43.5%), hepatocellular carcinoma (43.5%), primary or secondary sclerosing cholangitis (8.7%), or primary biliary cirrhosis (4.3%). The median model of end-stage liver disease (MELD) score was 13 (range, 6–38). Two patients underwent another LT within 48 h, one of whom had primary non-function, while the other had hepatic artery thrombosis (Table S1). The follow-up duration was 12 months, and 1 patient died after 3 months due to sepsis after a duodenal perforation. Another 2 patients did not attend follow-ups after 6 months.

### 3.1. LCP Tac Dosing

Median LCP Tac doses of 6.00 (4.00–9.00, 4.00–10.00, and 4.00–8.00 mg in the 1st week, 2nd week, and 1 month post-LT, respectively), 3.00 (2.00–4.75 mg at 6 months post-LT), and 2.08 mg (1.50–3.50 ng/dL at 12 months post-LT) were given.

**Figure 1:**
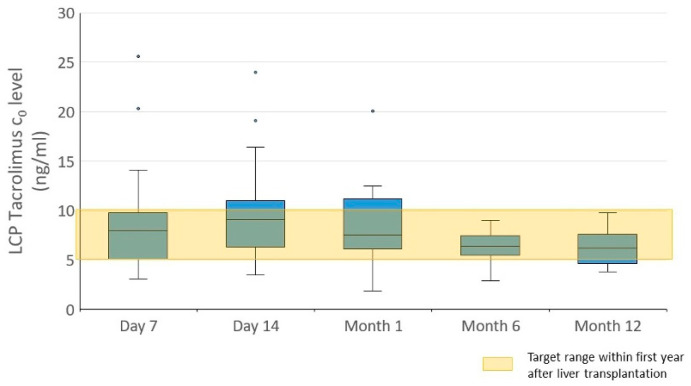
LCP Tacrolimus trough levels (LCP Tac_0_) at different time points within the first year after liver transplantation. The trough levels were significantly higher at 1 and 2 weeks than at 12 months (*p* = 0.030 and *p* = 0.042, respectively) and at 2 weeks than at 6 months (*p* = 0.038). The target level aimed for is indicated.

LCP Tac trough levels (LCP Tac_0_) were within the targeted range. Median LCP Tac_0_ serum levels of 8.00 (5.10–9.80), 9.1 (6.30–11.0), 7.50 (6.10–11.20), 6.40 (5.60–7.30), and 6.20 ng/mL (4.70–7.50) were determined in the 1st and 2nd week and at 1, 6, and 12 months post-LT, respectively. These included correspondingly decreasing values in the later course. LCP Tac_0_ differed significantly between the time points (*p* = 0.014). Its levels were significantly higher at 1 and 2 weeks post-LT when compared to 12 months post-LT (*p* = 0.030 and *p* = 0.042, respectively). Its levels at 2 weeks post-LT were also higher than at 6 months post-LT (*p* = 0.038) (Figure 1).

Median LCP Tac_peak_ serum levels of 15.10 (7.70–23.90), 9.1 (7.60–16.20), 16.9 (8.20–21.60), 7.70 (5.90–12.10), and 7.10 ng/mL (5.90–8.80) were determined in the 1st and 2nd week and at 1, 6, and 12 months post-LT, respectively.

No difference in LCP Tac_peak_ serum levels was found between the visits (*p* = 0.24). A proportion of 14.5% of the LCP Tac trough levels were in the subtherapeutic range, 69.1% were in the therapeutic range, and 16.4% were in the supratherapeutic range, with LCP Tac trough levels measured in all patients at all time points (week 1, week 2, month 1, month 6, year 1) (Figure S1, Table S2).

### 3.2. Cytokine Gene Expression Response

None of the 171 samples used for gene expression measurements were excluded due to methodological or technical problems (i.e., failed RNA extraction or immune activation, respectively).

**Figure 2:**
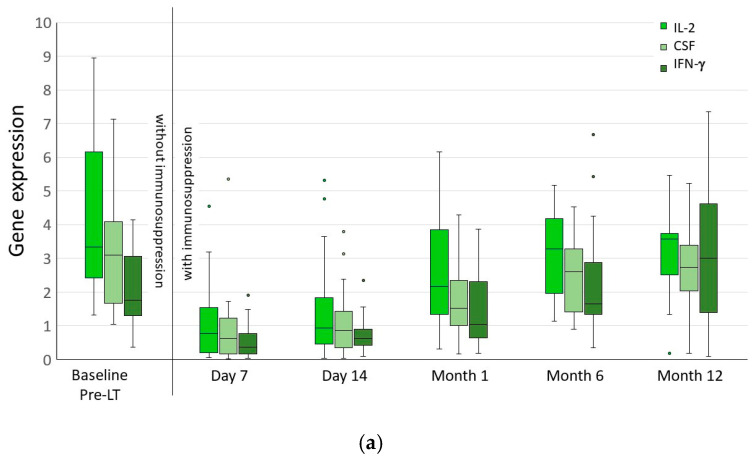

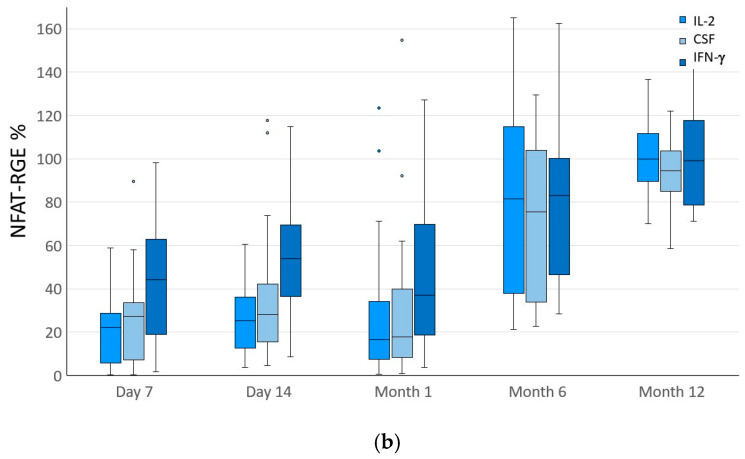
(**a**) Gene expression of IL-2, GM-CSF, and IFN-γ, measured before liver transplantation (c_pre-LT_) and after transplantation at the LCP Tacrolimus pre-dose level (c_0_). The expression levels of IL2, GM-CSF, and IFN-γ decreased from week 1 to month 1 and rose again to levels that nearly reached the pre-transplant level at months 6 and 12. Values are presented as medians (q1, q3). (**b**) Gene expression response calculated as the residual expression (RGE%) of IL-2, GM-CSF and IFN-γ after LCP Tacrolimus intake. After liver transplantation, the residual expression of NFAT-regulated genes after LCP Tac intakes was calculated as c_peak_/c_0_ × 100, with c_0_ as the number of transcripts at the LCP Tac pre-dose level and c_peak_ as the number of transcripts at the LCP Tac peak level. The strongest suppression of NFAT-regulated genes was observed until the first month after liver transplantation, with high residual NFAT-RGE levels seen after 6 months and 1 year. Values are presented as medians (q1, q3). The circles indicate values of outliers.

The NFAT-RGE response, including normalized copy numbers and calculated RGE%, changed significantly with time after LCP Tac intakes. The gene expression levels of IL-2, GM-CSF, and IFN-γ pre- and post-LT, at Tac’s pre-dose and peak levels, as RGE%, are expressed over time in Table 1 and Figure 2a,b. Values are expressed as medians (q1,q3).

The expression of IL-2, GM-CSF, and IFN-γ decreased from 1 week to 1 month post-LT and increased to near pre-transplant levels at 6 and 12 months post-LT.

There was no correlation between LCP Tac_0_ serum levels and RGE% at any study time point.

**Figure 3:**
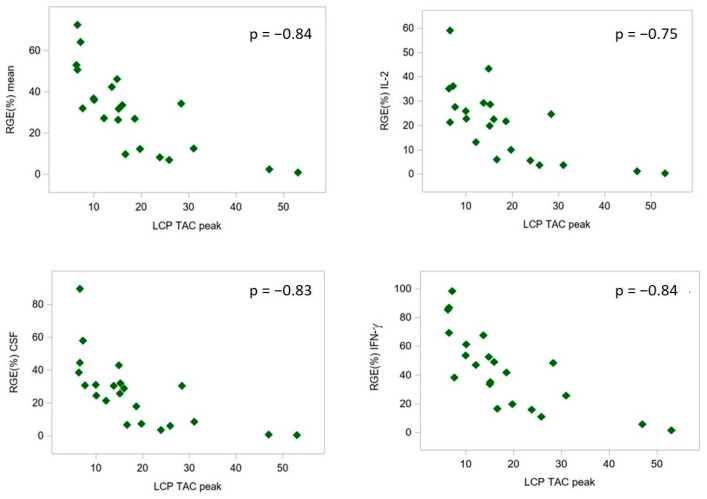
Relationship between LCP Tac_peak_ serum levels and representative residual expressions (RGE%) of IL-2, GM-CSF, and IFN-γ at day 7 after liver transplantation. A strong inverse correlation of the RGE% of each of the three genes with LCP Tac_peak_ serum levels was observed. The green symbols indicate the relationship between RGE% and LCP Tacp_eak_ values.

LCP Tac_peak_ serum levels and RGE% showed an overall strong inverse correlation at all time points analyzed (ρ = −0.8), as described in Table 2. This was also confirmed using dose-normalized LCP-Tac_peak_ concentrations adjusted to the patients’ body weight (Tables S3 and S4). Figure 3 representatively displays the relationship between the LCP Tac_peak_ serum levels and RGE% detected at 1 week post-LT.

### 3.3. Ranges of Residual Gene Expression with Respect to Clinically Significant Events

#### 3.3.1. Infection and Rejection

**Figure 4:**
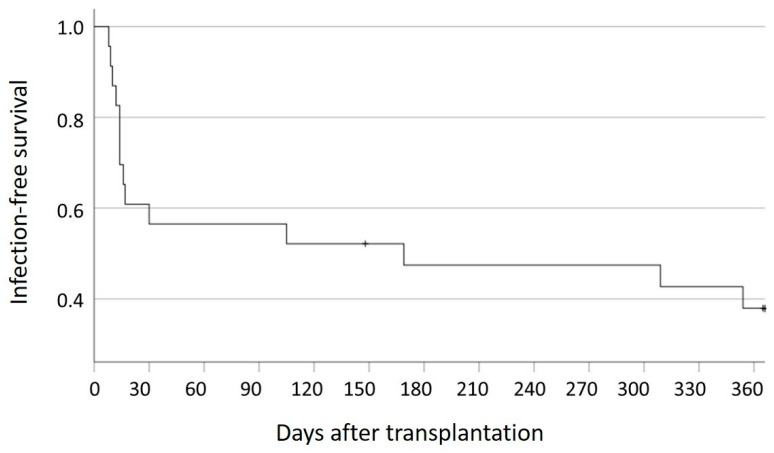
Infections after liver transplantation (LT) and infection-free survival. In 61% (n = 14) of patients, an infection was observed at a median of 169 days after liver transplantation. Eight patients did not experience an infection within the first year after transplantation. + One patient was lost to follow-up after 148 days.

An infection was observed after a median of 169 days post-LT in 61% (n = 14) of patients (Figure 4). The residual gene expression in individuals at the time of infection and in individuals without infection is shown in Table S5, and infection details are shown in Table S6. The median residual gene expression of NFAT-RGE in stable LT patients without infection 1 year post-LT was 94.25% (86.63–108.32). LT patients had 32.00% (23.30–95.10%) NFAT-RGE% at the time of their first infection post-LT (optional time point).

Based on our data and the corresponding existing literature, the optimal NFAT-RGE% range was between 25 and 50% within the first 6 months post-LT (Table 1 and Figure 2b). In the optimal NFAT-RGE% range, we observed infections in four patients, while seven infections were observed in patients who did not meet the optimal NFAT-RGE% range of 25–50%. The NFAT-RGE% values of three patients could not be calculated. The LCP Tacrolimus serum levels seen at the time of infection within the first year after liver transplantation are shown in Figure S2.

Only five rejections were observed, none of which occurred within the optimal NFAT RGE% range.

#### 3.3.2. Individual Immunosuppression Described by Individual LCP Tac Trough and Peak Effects (Effect LCP Tac_0_, Effect LCP Tac_peak_)

We assessed the individual effects of LCP Tac on the risk of infection. Medians of the individual trough and peak effects, pooled across the IL-2, GM-CSF, and IFN-γ levels at different time points post-LT, are shown in Table S7. The weakest effects were observed in the later stages (12 months post-LT).

**Figure 5:**
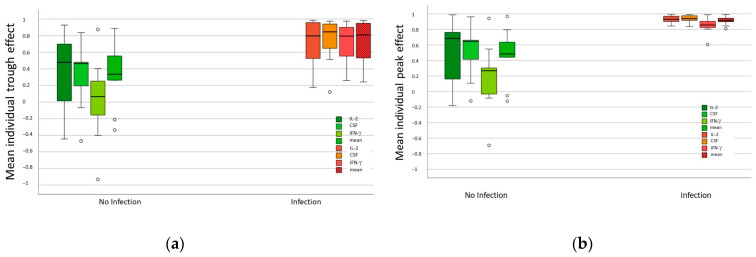
Mean individual trough and peak effects of LCP Tacrolimus in patients with infection and without infection: (**a**) The mean individual trough effects of each of the three NFAT-regulated genes, pooled over all measured time points, are shown. The through effect of LCP Tac (Effect LCP Tac_0_) was determined using the number of copies before LCP Tac intake (c_0_) for the respective visit, adjusted to the copies measured before liver transplantation (c_pre-LT_). Trough effects were significantly higher in patients with infection compared to patients without infection, with IFN-γ showing the greatest difference of the 3 genes. (**b**) The mean individual peak effects of each of the three NFAT-regulated genes, pooled over all measured time points, are shown. The peak effect of LCP Tac (Effect LCP Tac_peak_) was determined using the number the copies measured after LCP Tac intake at its peak levels (c_peak_) for the respective visit, adjusted to the number of copies measured before liver transplantation (c_pre-LT_). The peak effects of the 3 NFAT-regulated genes were significantly higher in patients with infections than in those without infections. IFN-γ showed the greatest difference of the 3 genes. The circles indicate values of outliers.

The mean individual trough and peak effects of each of the three NFAT-regulated genes, pooled over all time points, are shown in Table 3 and displayed in Figure 5a,b. The trough and peak effects of the three NFAT-regulated genes were significantly higher in patients with infections than in those without. Additionally, IFN-γ showed the most significant difference of the three genes.

## 4. Discussion

The present study investigates the feasibility of measuring NFAT-RGE in LT patients receiving extended-release LCP Tac during their first year post-operation, focusing on over-IS and infection.

We found a strong inverse correlation between the LCP Tac_peak_ serum level and the weight-adjusted LCP-Tac_peak_ concentration and the residual expression of NFAT-regulated genes. The significantly lower infection rate observed in patients with a high NFAT-RGE% range suggests that immunosuppression based on NFAT-RGE measurements could prevent infections associated with over-immunosuppression early after LT.

This is the first study to clearly confirm the feasibility and safety of the present diagnostic methods in patients on IS with LCP Tac. Thus, the proof of concept was realized successfully. Considering the relationship between drug levels and residual NFAT-RGE, we will be able to identify patients with excessive IS. This will allow us to reduce the LCP Tac dose while monitoring the relevant genes’ expression. The main objective of further studies should be to develop a tailor-made IS regime for long-term stable LT patients based on the determination of their residual NFAT-RGE.

When reviewing the literature on NFAT-RGE after cyclosporine A, 10 studies comprising 702 patients after kidney transplantation (KT) [9,12,13,14,15,16,17,18,19,20] and 5 studies containing 153 patients after LT [21,22,23,24,25] were investigated. Of these, two studies included pediatric patients (KT, n = 1; LT, n = 1) and another two studies, comprising 79 heart transplant patients, were reviewed [9,26]. We have found that most of these studies were conducted in patients with long-term stability. There is only one de novo study on the impact of NFAT-RGE in 80 patients after KT. While most trials were prospective, observational, or cross-sectional, only one prospective randomized controlled trial (RCT) was found. In another study, NFAT-RGE and the monitoring of CNI trough levels were compared in 55 stable patients after KT [13]. Most studies have shown an association between high NFAT-RGE levels and rejection episodes after LT, whereas low NFAT-RGE levels have been associated with increased infection rates (Table 4).

Few studies have investigated NFAT-RGE after twice-daily Tacrolimus (Tac) treatments. We found four studies, for a total of 428 Tac-treated patients, where long-term stable patients were investigated after KT [11,27,28,29], two studies including de novo KT patients [30,31], and one RCT. The RCT compared NFAT-RGE measurements with CNI trough level monitoring in 40 stable patients after KT [32]. We found four further studies in which 130 patients given Tac were monitored after LT [21,22,24,25,33] (Table 4).

A study by Bremer et al. [30] was the only one to report NFAT-RGE after once-daily Tac treatment. However, 19 of the 29 patients had been switched from the regular twice-daily formulation of Tac (Prograf^®^) to a once-daily formulation (Advagraf^®^) at 1 year post-KT.

According to the literature published in the field, NFAT-RGE measurements might be an optimized way of monitoring and individualizing the level of IS achieved with CNIs. An NFAT-RGE analysis is relatively specific for Tac exposure [9]. As outlined above, most published studies have focused on long-term stable patients, mainly after KT, and two randomized controlled trials on KT patients have been published to date [13,32]. Only 19 cases of once-daily Tac administration in KT patients and NFAT-RGE monitoring 1 year after KT have been reported to date [30].

In our cohort, patients showed the strongest suppression of NFAT-RGE when their residual NFAT-RGE was equivalent to low copy numbers of the three target genes early post-LT, and these numbers increased again after 6 and 12 months. This is in accordance with the published literature. Bremer et al. reported higher residual NFAT-RGEs observed at 1 year on once-daily Tac versus 1 week post-KT with twice-daily Tac, which was suspected to not be explained by the switch in formulations. However, the authors emphasized the need for further investigation of NFAT-RGE during treatment with once-daily formulations in view of the increasing use of extended-release formulations [30].

A descriptive analysis of our data confirmed that although patients exhibited an LCP Tac trough level within the targeted therapeutic window, a low NFAT-RGE% serum level could result in a higher risk of infection. Based on our data and the corresponding existing literature, the optimal NFAT-RGE% range during the first 6 months after LT is between 25 and 50% [21,22,24,25,33]. Within that range, 43% fewer infections were recorded and no rejections were observed. Outside the optimal range, there were rejections in 19% of the patients. We also assumed a lower tumor recurrence rate, which is of the utmost importance in the face of almost 50% of the patients in our cohort having a malignant disease (hepatocellular carcinoma) before transplantation. An observation period longer than 1 year would be recommended for future studies.

The strengths of the present study are in its prospective design, following patients post-LT, with precisely predefined time points for control visits and a uniform immunosuppressive regimen in all patients. For the first time, we were able to take the immune status of each patient before IS into account, determining the individual peak and trough effects of LCP Tac by referring to each patient’s NFAT status pre-LT.

Concerning the individual immunosuppressive status of all patients before transplantation, the individual trough and peak effects of LCP Tac at defined time points post-LT differed significantly between patients with and without infection. In contrast, there was no difference in LCP Tac’s trough and peak serum levels. Although only 23 patients were included in our trial, there were significant results within our study, and the primary endpoint was met.

Some limitations of the present study should be kept in mind. As a single-center study, the number of patients included were limited. Therefore, further investigations of different infection types were not possible. Due to the occurrence of heterogeneous infections of unequal severity, the results reported in this proof of concept study have to be interpreted with caution.

Further studies should focus on separate analyses of different types of infections, such as opportunistic infections. Also, a possible relationship between individual LCP-Tac serum levels within different therapeutic ranges and the occurrence of infections needs to be examined in studies with a larger sample size.

In summary, a strong inverse correlation between the LCP Tac_peak_ serum level and the residual expression of NFAT-regulated genes was found, indicating that the quantitative analysis of these genes enables reliable biological IS at the target cell level in de novo LT recipients. There was a difference between NFAT-RGE% in patients with and without infection, which was significant when considering the individual status of IS in each patient pre-LT. In the optimal NFAT-RGE% range, the rates of infection and rejection decreased distinctly, by 43 and 100%, respectively.

This is the first study to clearly confirm both the feasibility and safety of the present diagnostic methods in patients undergoing IS with LCP Tac, and the proof of concept was successfully realized. Considering the relationship between the drug’s levels and residual NFAT-RGE, we will be able to identify patients with excessive IS. This will allow us to reduce the LCP Tac dose while monitoring the relevant gene expression. The main objective of further studies should be to develop a tailor-made IS for long-term stable LT patients based on determining their residual NFAT-RGE.

## Figures and Tables

**Table 1:** Gene expression of IL2, GM-CSF, and IFN-γ prior to liver transplantation (pre-LT) and prior to LCP Tacrolimus intake (c_0_) at different time points. Gene expression of IL2, GM-CSF, and IFN-γ after LCP Tacrolimus intake (c_peak_) at different time points after liver transplantation. Residual gene expression (RGE%) of IL2, GM-CSF, and IFN-γ after LCP Tacrolimus intake at different time points after liver transplantation.

Normalized Copies	Time Point	Median	q1	q3
IL2 c_0_	Pre-LT (cpre-LT)	3.34	2.43	6.16
	Week 1	0.76	0.21	1.53
	Week 2	0.93	0.46	1.84
	Month 1	2.16	1.33	3.86
	Month 6	3.29	2.01	4.17
	Year 1	3.58	2.52	3.73
CSF c_0_	Pre-LT (cpre-LT)	3.10	1.67	4.08
	Week 1	0.62	0.16	1.22
	Week 2	0.86	0.35	1.42
	Month 1	1.53	1.00	2.35
	Month 6	2.61	1.63	3.25
	Year 1	2.73	2.05	3.39
IFN-γ c_0_	Pre-LT (cpre-LT)	1.76	1.31	3.06
	Week 1	0.36	0.16	0.77
	Week 2	0.63	0.42	0.90
	Month 1	1.04	0.64	2.32
	Month 6	1.64	1.37	2.71
	Year 1	3.01	1.42	4.45
IL2 c_peak_	Week 1	0.10	0.03	0.30
	Week 2	0.25	0.14	0.31
	Month 1	0.37	0.11	0.89
	Month 6	3.17	1.25	4.38
	Year 1	2.98	2.55	3.83
CSF c_peak_	Week 1	0.10	0.03	0.27
	Week 2	0.15	0.14	0.40
	Month 1	0.32	0.10	0.63
	Month 6	2.14	1.22	2.67
	Year 1	2.57	2.18	3.15
IFN-γ c_peak_	Week 1	0.15	0.03	0.40
	Week 2	0.34	0.17	0.50
	Month 1	0.36	0.15	1.01
	Month 6	1.23	0.90	2.40
	Year 1	2.90	1.71	4.03
RGE% IL2	Week 1	22.07	28.50	6.03
	Week 2	25.20	35.37	13.11
	Month 1	16.63	33.36	8.73
	Month 6	81.50	112.93	40.12
	Year 1	100.05	111.65	89.54
RGE% GM-CSF	Week 1	27.38	31.79	7.36
	Week 2	28.23	41.79	15.82
	Month 1	17.75	36.96	9.87
	Month 6	75.62	101.30	39.94
	Year 1	94.35	103.59	85.14
RGE% IFN-γ	Week 1	44.31	61.23	19.65
	Week 2	54.13	62.82	38.68
	Month 1	36.92	68.86	19.18
	Month 6	83.06	99.79	46.83
	Year 1	98.97	117.55	78.67

**Table 2:** Residual gene expression (RGE%) of IL2, GM-CSF, and IFN-γ and their correlation with LCPTac_peak_ serum level at different time points after liver transplantation.

Time Point	Gene	RGE% (Median)	Median TAC_peak_ Serum Level (ng/mL)	Correlation Coefficient (ρ)
Week 1	IL2	22.07		−0.753
	GM-CSF	27.38	15.1	−0.827
	IFN-γ	44.31		−0.843
Week 2	IL2	25.20		−0.520
	GM-CSF	28.23	9.1	−0.728
	IFN-γ	54.13		−0.505
Month 1	IL2	16.63		−0.803
	GM-CSF	17.75	16.9	−0.828
	IFN-γ	36.92		−0.730
Month 6	IL2	81.50		−0.808
	GM-CSF	75.62	7.7	−0.647
	IFN-γ	83.06		−0.557
Year 1	IL2	100.05		−0.320
	GM-CSF	94.35	7.1	−0.190
	IFN-γ	98.97		−0.313

**Table 3:** Individual immunosuppression (Effect LCP Tac_peak_, Effect LCP Tac_0_) of different NFAT-regulated genes pooled across all time points.

Gene	Mean Individual Trough Effect in Patients			Mean Individual Peak Effect in Patients		
	without Infection	with Infection	*p*	without Infection	with Infection	*p*
IL2	0.48 (0.02–0.70)	0.80 (0.53–0.96)	<0.023	0.68 (0.16–0.76)	0.93 (0.90–0.97)	<0.004
GM-CSF	0.46 (0.20–0.48)	0.85 (0.65–0.94)	<0.001	0.65 (0.42–0.66)	0.94 (0.92–0.98)	<0.001
IFN-γ	0.07 (−0.16–0.25)	0.79 (0.55–0.90)	<0.001	0.27 (−0.03–0.30)	0.86 (0.82–0.90)	<0.001
All 3 genes	0.33 (0.26–0.56)	0.81 (0.53–0.95)	<0.011	0.48 (0.44–0.64)	0.91 (0.90–0.94)	<0.001

**Table 4:** Clinical studies on the quantitative analysis of NFAT-regulated gene expression in the peripheral blood of solid organ recipients on Cyclosporine A therapy. Clinical studies on the quantitative analysis of NFAT-regulated gene expression in the peripheral blood of solid organ recipients on Tacrolimus therapy.

Reference	Study Design	Patient Cohort	Main Result
Sommerer et al. [12]	Prospective observational	Kidney (n = 176)	Low NFAT-RGE proven as risk factor for BK viremia.
Sommerer et al. [14]	Prospective observational	De novo kidney (n = 80)	Low NFAT-RGE associated with infectious complications and high NFAT-RGE associated with risk of rejection.
Sommerer et al. [15]	Prospective observational	Senior kidney (n = 36)	Low NFAT-RGE associated with opportunistic infections.
Sommerer et al. [16]	Prospective observational	Kidney (n = 133)	Low NFAT-RGE is associated with infections and malignancy.
Giese et al. [9]	Cross-sectional	Kidney (n = 25), liver (n = 14), heart (n = 26)	Pilot study to assess the property of the assay; PK–PD analysis. NFAT-RGE as marker for individual responsiveness.
Sommerer et al. [17]	Prospective observational case–control	Kidney (n = 20), matched controls (n = 20)	Tapering of cyclosporin A, monitored by RGE, improved allograft function and reduced cardiovascular risk factors.
Sommerer et al. [18]	Prospective observational	Senior kidney (n = 55)	Low NFAT-RGE associated with nonmelanoma skin cancer.
Dannewitz et al. [19]	Prospective observational	Kidney (n = 144)	Low NFAT-RGE associated with cyclosporine A in saliva and gingival hyperplasia.
Sommerer et al. [13]	Prospective interventional randomized-controlled	Kidney (n = 55)	NFAT-RGE leads to an improved cardiovascular risk profile and improves long-term renal allograft function.
Billing et al. [20]	Cross-sectional observational	Pediatric kidney (n = 45)	Low NFAT-RGE is associated with infection episodes.
Billing et al. [21]	Prospective observational	Pediatric liver (n = 33)	Low NFAT-RGE in recurrent infection.
Zahn et al. [22]	Cross-sectional	Liver (n = 45)	Applicability of NFAT-RGE monitoring in liver transplantation confirmed.
Herden et al. [23]	Prospective observational	Liver (n = 20 long-term, n = 20 de novo)	NFAT-RGE correlated with infection parameters.
Steinebrunner et al. [24]	Prospective observational	Liver (n = 13)	High NFAT-RGE in acute rejection.
Steinebrunner et al. [25]	Cross-sectional	Liver (n = 10)	Low NFAT-RGE in CMV infection.
Giese et al. [9]	Cross-sectional	Kidney (n = 25), liver (n = 14), heart (n = 26)	Pilot study to assess the property of the assay; PK–PD analysis. NFAT-RGE as marker for individual responsiveness.
Konstandin et al. [26]	Prospective observational	Heart (n = 53)	Low NFAT-RGE is associated with recurrent infections.
Sommerer et al. [27]	Cross-sectional	Kidney (n = 262)	PK–PD correlation, low NFAT-RGE is associ-ated with recurrent infections and high NFAT-RGE with rejection.
Sommerer et al. [28]	Prospective observational	Kidney (n = 73)	Low NFAT-RGE is associated with CMV viremia.
Sommerer et al. [29]	Prospective observational	Kidney (n = 23)	Low NFAT-RGE as risk factor for BKV viremia.
Keller et al. [11]	Prospective observational	Kidney (n = 10)	Pharmacodynamics of Tac described in dependence of pharmakokinetics.
Bremer et al. [30]	Prospective	Kidney (n = 29)	Potential of NFAT-RGE as a tool for the pharmacodynamic monitoring of Tac therapy in the early phase post-Tx, especially in the context of over-immunosuppression and viraemia confirmed.
Sommerer et al. [31]	Prospective observational	De novo kidney (n = 60)	NFAT-RGE was confirmed as a potential noninvasive early predictive biomarker in the immediate post-transplant period to detect patients at risk of acute rejection and infectious complications in Tac-treated renal allograft recipients.
Webber et al. [32]	Randomized Controlled Pilot Trial	Kidney (n = 40)	Quantitative analysis of nuclear factor of activated T-regulated gene expression may serve as a reliable assay to lower tac dosing.
Billing et al. [21]	Prospective observational	Pediatric liver (n = 33)	Low NFAT-RGE in recurrent infection.
Zahn et al. [22]	Cross-sectional	Liver (n = 45)	Applicability of NFAT-RGE monitoring in liver transplantation confirmed.
Steinebrunner et al. [24]	Prospective observational	Liver (n = 13)	High NFAT-RGE in acute rejection.
Steinebrunner et al. [25]	Cross-sectional	Liver (n = 10)	Low NFAT-RGE in CMV infection.
Millán et al. [33]	Prospective observational	Liver (n = 56)	Sequential post-transplantation NFAT-RGE monitoring combined with intralymphocytary IL-2 and IFN-γ may be key predictive and diagnostic biomarkers of the risk of TCMAR and SCR and better guide CNi therapy in LT patients.

## Data Availability

All of the analyzed data are contained within the article or Supplementary Material. The raw data on gene expression are available on request from the corresponding author.

## Supplementary Materials

The following supporting information can be downloaded at: https://www.mdpi.com/article/10.3390/pharmaceutics16101317/s1. Table S1: Patient characteristics. Table S2: Results of LCP Tacrolimus trough level measurements, categorized by different therapeutic ranges. Table S3: Residual gene expression (RGE%) of IL2, GM-CSF, and IFN-γ and their correlation with LCP Tac_peak_ concentration, adjusted to patient’s body weight at different time points after liver transplantation. Table S4: LCP Tac_0_ and LCP Tac_peak_ concentration, adjusted to patient’s body weight at different time points after liver transplantation. Table S5: Descriptive statistics of RGE IL-2, RGE GM-CSF, RGE IFN-γ, and RGE mean at the time of infection and without infection. Table S6: Infection details. Table S7: Individual immunosuppression (Effect LCP Tac_peak_, Effect LCP Tac_0_), pooled across IL-2, GM-CSF, and IFN-γ, after liver transplantation. Figure S1: LCP Tacrolimus trough levels, categorized by different therapeutic ranges. Figure S2: LCP Tacrolimus serum levels at the time of infection within the first year after liver transplantation.
