# Supplementary material for: Use of Nuclear Factor of Activated T Cell-Regulated Gene Expression for Monitoring Immunosuppression with Extended-Release Tacrolimus after Liver Transplantation—A Proof of Concept
Source: Pharmaceutics. 2024 Oct 11;16(10):1317. doi: 10.3390/pharmaceutics16101317 (PMC11511070; doi:10.3390/pharmaceutics16101317)
Supplement: Supplementary file 1 [file pharmaceutics-16-01317-s001.zip › pharmaceutics-3210133-Supplementary.pdf]

pharmaceutics

MDPI

Article

Supplementary Materials: Use of Nuclear Factor of Activated T Cell-Regulated Gene Expression for Monitoring Immunosuppression with Extended-Release Tacrolimus after Liver Transplantation—A Proof of Concept

Judith Kahn, Eva Maria Matzhold, Peter Schlenke and Peter Schemmer

Table S1. Patient characteristics.

Characteristics	Statistics
	(n = 23)
**Sex**	
Male	15 (65.22%)
Female	8 (34.78%)
**Age at time of LT (y)**	58.39 ± 10.50
**Aetiology of liver disease**	
HCC	10 (43.48%)
Alcoholic liver disease	10 (43.48%)
Other	3 (13.04%)
**MELD score**	13.83 ± 7.43

LT: Liver transplantation; HCC: Hepatocellular carcinoma; MELD: Model of end-stage liver disease.

Table S2. Results of LCP Tacrolimus trough level measurements categorized by different therapeutic ranges.

LCP Tac0 levels	Week 1 (N)	Week 2 (N)	Month 1 (N)	Month 6 (N)	Year 1 (N)
Subtherapeutic range	4	2	2	2	6
Therapeutic range	14	15	14	19	14
Supratherapeutic range	5	6	7	0	0

Results of LCP Tac through level analyzes (LCP Tacrolimus c_0) as performed in all study patients at different time-points after liver transplantation are summarized.
Subtherapeutic range: LCP Tacrolimus c_0 level < 5 ng/ml);
Therapeutic range: LCP Tacrolimus c_0 level 5–10 ng/ml;
Supratherapeutic range: LCP Tacrolimus c_0 level > 10 ng/ml;
N = Number of LCP Tac trough level analyzes.

Table S3. Residual gene expression (RGE%) of IL2, GM-CSF and IFN-γ and correlation with LCP Tac_{peak} concentration adjusted to body weight at different time points after liver transplantation.

Time point	Gene	RGE% (Median)	Median LCP TAC_{peak} concentration adjusted to body weight (μg l^{-1})/(mg kg^{-1})	Correlation coefficient (ρ)
Week 1	IL2	22.07		−0.526
	GM-CSF	27.38	175.58	−0.637
	IFN-γ	44.31		−0.648
Week 2	IL2	25.20		−0.477
	GM-CSF	28.23	135.72	−0.325

	IFN-γ	54.13		−0.224
Month 1	IL2	16.63		−0.335
	GM-CSF	17.75	196.35	−0.430
	IFN-γ	36.92		−0.385
Month 6	IL2	81.50		−0.365
	GM-CSF	75.62	253.5	−0.249
	IFN-γ	83.06		−0.280
Year 1	IL2	100.05		−0.223
	GM-CSF	94.35	290.4	−0.163
	IFN-γ	98.97		−0.437

LCP Tac$_{peak}$ concentration adjusted to the patients′ body weight and RGE% showed an strong inverse correlation at all time points analyzed.

Table S4. LCP Tac$_{0}$ and LCP Tac$_{peak}$ concentration adjusted to body weight at different time points after liver transplantation.

Variable	N	Median	Lower quartile	Upper quartile	Minimum	Maximum
LCP Tac$_{0\ adjusted}$	22	115.43	52.50	137.14	21.70	376.00
LCP Tac$_{0\ adjusted}$	22	175.58	131.30	277.22	40.95	446.04
BW (kg)	22	75.00	63.00	83.00	52.00	99.00
Dose LCP Tac$_{peak}$	23	6.00	5.00	10.00	0.96	13.00
Dose LCP Tac$_{0}$	23	6.00	4.00	9.00	0.96	14.00
LCP Tac$_{0\ adjusted}$	22	102.46	63.75	166.00	36.54	1116.63
LCP Tac$_{0\ adjusted}$	21	135.72	78.00	226.10	34.02	1036.05
BW (kg)	23	75.00	63.00	83.00	52.00	99.00
Dose LCP Tac$_{peak}$	23	5.00	3.00	8.00	0.72	14.00
Dose LCP Tac$_{0}$	23	6.00	4.00	10.00	0.00	14.00
LCP Tac$_{0\ adjusted}$	23	91.87	56.86	161.85	26.46	616.09
LCP Tac$_{0\ adjusted}$	23	196.35	104.08	286.35	30.00	600.49
BW (kg)	23	75.00	63.00	83.00	52.00	99.00
Dose LCP Tac$_{peak}$	23	7.00	4.00	8.00	0.87	13.00
Dose LCP Tac$_{0}$	23	6.00	4.00	8.00	0.87	15.00
LCP Tac$_{0\ adjusted}$	21	146.85	105.00	253.15	54.25	376.30
LCP Tac$_{0\ adjusted}$	21	253.50	141.44	418.90	47.60	677.60
BW (kg)	23	75.00	63.00	83.00	52.00	99.00
Dose LCP Tac$_{peak}$	21	3.00	2.00	4.75	1.00	6.00
Dose LCP Tac$_{0}$	21	3.00	2.00	4.75	1.00	6.00
LCP Tac$_{0\ adjusted}$	20	200.25	149.28	274.87	51.52	580.00
LCP Tac$_{0\ adjusted}$	19	290.40	197.60	390.50	79.52	790.00
BW (kg)	23	75.00	63.00	83.00	52.00	99.00
Dose LCP Tac$_{peak}$	20	2.00	1.50	3.00	0.75	5.00
Dose LCP Tac$_{0}$	20	2.08	1.50	3.50	0.75	5.00

LCP Tac$_{0}$ concentration adjusted to body weight (LCP Tac$_{0\ adjusted}$) (µg l–1)/(mg kg–1);.
LCP Tac$_{peak}$ concentration adjusted to body weight (LCP Tac$_{0\ adjusted}$) (µg l–1)/(mg kg–1);.
BW (body weight) (kg);.
Dose LCP Tac$_{0}$ (mg) given before LCP Tac$_{0}$ level measurement;.
Dose LCP Tac$_{peak}$ (mg) given before LCP Tac$_{0}$ level measurement;.
N: Number of analyzes performed;.

Table S5. Descriptive statistics of RGE IL-2, RGE GM-CSF, RGE IFN-γ, RGE mean in individuals at the time of infection and without infection.

	Infection	n	Median	min	max	q1	q3
RGE IL-2	0	8	94.1	71.3	136.6	90.95	123.55
	1	11	28.6	10.0	112.9	15.80	60.70
RGE GM-CSF	0	8	91.1	58.7	107.2	75.65	102.70
	1	11	28.5	15.8	117.8	17.80	114.10
IFN-γ	0	8	92.2	77.5	133.8	82.45	108.75
	1	11	41.6	25.0	114.4	34.20	99.80
RGE mean	0	8	94.3	71.2	119.7	86.63	108.32
	1	11	32.0	20.4	114.4	23.30	95.10

Fourteen study patients suffered from infection. Eight patients had never experienced an infection within the first year. RGE values of 4 patients (3 with infection, 1 without infection) couldn´t be determined.
0: No Infection; 1: with Infection;.

Table S6. Infection details.

Infection details	Statistics
	(n = 23)
**Infection**	14 (60.8%)
Sepsis/death	2 (8.7%)
Bacterial/fungal	11(47.8%)
Viral/CMV	5 (27.7%)

Table S7. Individual immunosuppression (Effect LCP Tacpeak, Effect LCP Tac0) pooled across IL-2, GM-CSF and IFN- expression after liver transplantation.

	Patients without infection		Patients with infection	
	Median individual trough effect	Median individual peak effect	Median individual trough effect	Median individual peak effect
Week 1	0.78 (0.59–0.94)	0.96 (0.90–0.98)	N.A.	N.A.
Week 2	0.57 (0.38–0.77)	0.91 (0.81–0.93)	0.91 (0.64–0.96)	0.92 (0.92–0.92)
Month 1	0.20 (−0.05–0.40)	0.84 (0.78–0.93)	0.81 (0.81–0.81)	0.88 (0.88–0.88)
Month 6	−0.01 (−0.40–0.38)	0.26 (−0.12–0.54)	N.A.*	N.A.*
Year 1	−0.11 (−1.12–0.02)	−0.05 (−0.67–0.05)	N.A.*	N.A.*

N.A. Not applicable; No infection was observed during the first week.
* Only 1 infection occurred during the time period.

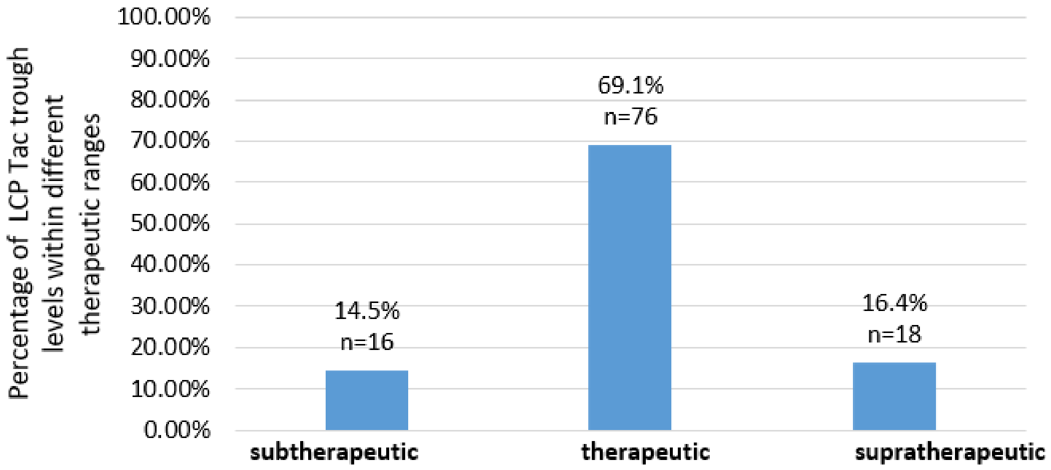

Figure S1. LCP Tacrolimus trough levels categorized by different therapeutic ranges.

Results of LCP Tac through level analysis (LCP Tacrolimus c_0) as performed in all study patients at different time-points after liver transplantation are summarized.
Subtherapeutic range: LCP Tacrolimus c_0 level < 5 ng/ml;
Therapeutic range LCP Tacrolimus c_0 level 5–10 ng/ml;
Supratherapeutic range: LCP Tacrolimus c_0 level > 10 ng/ml;
n = Number of LCP Tac trough level analysis.

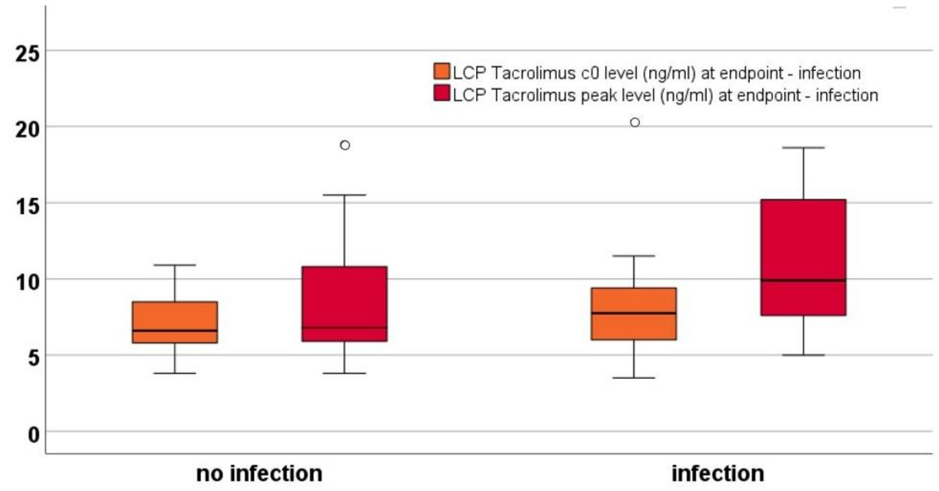

Figure S2. LCP Tacrolimus serum levels at the time of infection within the first year after liver transplantation. Trough (LCP Tacrolimus c_0) and peak levels (LCP $Tacrolimus_{peak}$) of individuals with infection compared to values of individuals without infection are shown. The circles indicate values of outliers.
